# TRIM Proteins: Key Regulators of Immunity to Herpesvirus Infection

**DOI:** 10.3390/v16111738

**Published:** 2024-11-06

**Authors:** Zuberwasim Sayyad, Dhiraj Acharya, Michaela U. Gack

**Affiliations:** Florida Research and Innovation Center, Cleveland Clinic, 9801 SW Discovery Way, Port St. Lucie, FL 34987, USA; sayyadz@ccf.org

**Keywords:** herpesvirus, HSV-1, innate immunity, TRIM proteins, cGAS, STING, RIG-I-like receptors, Toll-like receptors, autophagy, apoptosis

## Abstract

Herpesviruses are ubiquitous DNA viruses that can establish latency and cause a range of mild to life-threatening diseases in humans. Upon infection, herpesviruses trigger the activation of several host antiviral defense programs that play critical roles in curbing virus replication and dissemination. Recent work from many groups has integrated our understanding of TRIM (*tripartite motif*) proteins, a specific group of E3 ligase enzymes, as pivotal orchestrators of mammalian antiviral immunity. In this review, we summarize recent advances in the modulation of innate immune signaling by TRIM proteins during herpesvirus infection, with a focus on the detection of herpes simplex virus type 1 (HSV-1, a prototype herpesvirus) by cGAS-STING, RIG-I-like receptors, and Toll-like receptors. We also review the latest progress in understanding the intricate relationship between herpesvirus replication and TRIM protein-regulated autophagy and apoptosis. Finally, we discuss the maneuvers used by HSV-1 and other herpesviruses to overcome TRIM protein-mediated virus restriction.

## 1. Introduction

### 1.1. Herpesviruses, Herpesvirus-Induced Diseases, and Clinical Importance

The *Herpesviridae* family is a large group of enveloped, double-stranded DNA (dsDNA) viruses that can infect vertebrates and invertebrates and are classified into alpha (α), beta (β), and gamma (γ) subfamilies based on their genomic architecture, replication strategies, and host range [1]. Of more than 130 herpesviruses identified so far, *Alphaherpesvirinae* subfamily members characterized by a relatively short replication cycle include herpes simplex virus type 1 and 2 (HSV-1 and HSV-2, also known as HHV-1 and HHV-2) and varicella-zoster virus (VZV or HHV-3). Human herpesviruses with a longer replicative cycle (of the *Betaherpesvirinae* subfamily) comprise human cytomegalovirus (HCMV or HHV-5), human herpesviruses 6A and 6B (HHV-6A and HHV-6B), and human herpesvirus 7 (HHV-7). The other two clinically important herpesviruses, Epstein–Barr virus (EBV or HHV-4) and Kaposi’s sarcoma-associated herpesvirus (KSHV, also known as HHV-8), are members of the *Gammaherpesvirinae* subfamily and have a quite restricted host range. Unique to herpesviruses (and distinct from many RNA viruses) is their ‘lytic-to-latent-to-lytic’ replication cycle, which allows these viruses to undergo lifelong persistent infection but can also cause occasional ‘reactivation’, the re-entering into the lytic phase after latency establishment [2].

Herpesviruses can cause a range of mild to life-threatening diseases including virus-induced cancers in humans [1]. HSV-1 and HSV-2 are neurotropic viruses that can cause oral or genital mucocutaneous lesions (most commonly) and encephalitis or meningitis (rarely). HSV-1 infections affect ~3.7 billion people (under the age of 50), while HSV-2 infections occur in ~490 million people aged between 15 and 49, thus representing a major health burden across the globe [3]. HSV-1 and HSV-2 initially infect skin and mucosal epithelial cells to carry out the lytic replication cycle, and eventually establish latency in sensory nerve ganglia where they remain throughout life [1,2,4]. Lytic HSV-1/2 infection of the skin causes cell damage leading to the formation of small painful, fluid-filled blisters at the site of infection. Moreover, HSV-1 can attack the cornea inducing keratitis, which can cause significant vision impairment or even blindness [5]. Herpes simplex encephalitis (HSE) is a rare disease caused by HSV-1/2 that can lead to severe brain inflammation and is associated with high mortality if untreated [6]. Among the clinically important herpesviruses is also HCMV, which causes mononucleosis syndrome with fever, malaise, lymphocytosis, and pharyngitis [1]. In addition, HCMV can cause congenital infection and life-threatening disseminated disease in immunocompromised hosts involving various organs such as the lungs, liver, gastrointestinal tract, retina, and/or the central nervous system (CNS). Primary VZV infection causes varicella (chickenpox), whereas reactivation of latent VZV (usually in adults) causes herpes zoster (shingles) [1]. EBV and KSHV are known to cause several malignancies in humans including primary effusion lymphoma and Kaposi’s sarcoma (both caused by KSHV) or Burkitt’s lymphoma, nasopharyngeal cancer, stomach cancer, Hodgkin lymphoma, and head and neck cancer (caused by EBV) [7,8,9].

Herpesvirus infections have also recently been implicated in neurodegenerative diseases or certain autoimmune conditions [10,11,12]. For example, several studies showed that EBV infection is causally linked to multiple sclerosis (MS) [13]. Similarly, HSV-1 and HHV-6 infections in the brain have been associated with neurological damage and other pathological hallmarks of Alzheimer’s disease (AD) [12,14,15]. However, additional investigations are necessary to gain a more comprehensive understanding of the detailed mechanisms underlying the potentially causal connection between herpesviruses and neurodegenerative diseases.

Acyclovir (ACV) and its derivatives are widely used to combat certain herpesvirus infections; however, extended treatment has led to ACV-resistant HSV strains that can be detrimental to immunocompromised patients [16,17,18]. For several other herpesviruses (e.g., EBV and KSHV), no effective specific antiviral therapeutics are currently available. On the other hand, genetically modified herpesviruses have recently been developed for treating certain types of cancer. For example, talimogene laherparepvec (T-VEC) is an engineered oncolytic HSV-1 variant approved for viral immunotherapy of melanoma [19,20]. Enhancing our molecular understanding of herpesviral interactions with specific human factors involved in the intrinsic or innate immune response could lead to novel strategies for developing anti-herpesviral therapeutics or may guide the design of improved oncolytic virotherapy.

### 1.2. Tripartite Motif-Containing Proteins: Critical Regulators of Virus Infection

**Figure 1:**
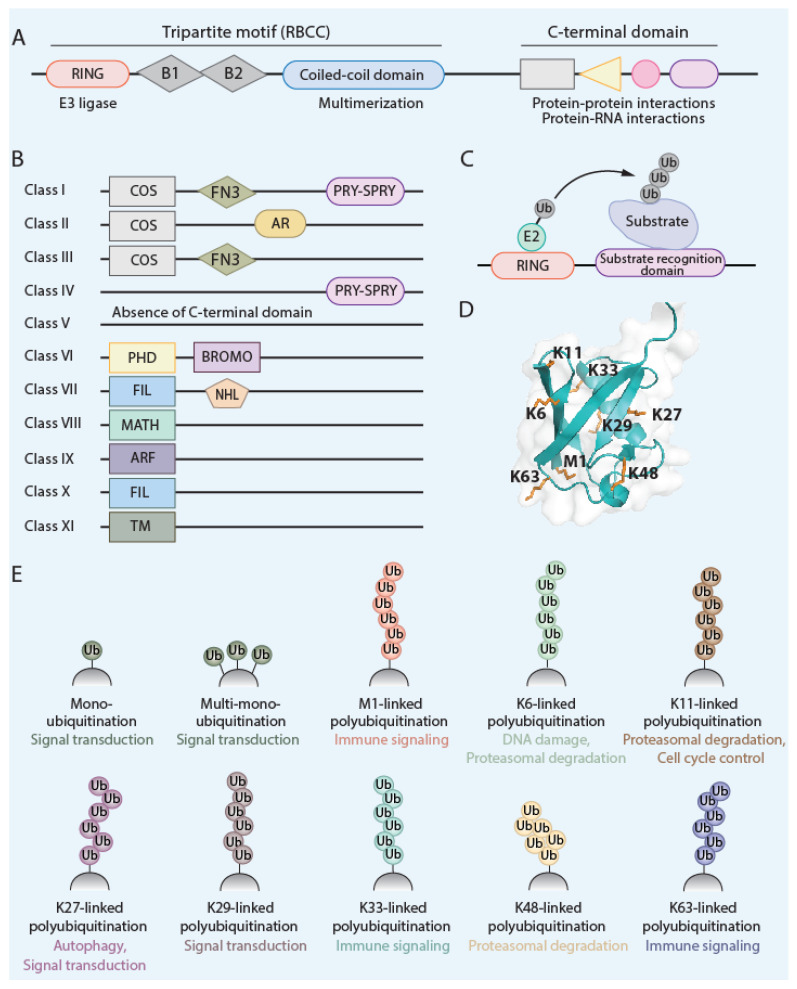
Domain architecture, subgroup classification, and ubiquitin E3 ligase activities of TRIM proteins: (**A**,**B**). The prototypical domain architecture of TRIM proteins consists of a RING domain, B Box domain(s), coiled-coil domain, and variable C-terminal domains (**A**). Based on the specific C-terminal variable region, TRIM proteins are classified into 11 distinct subgroups (**B**). The C-terminal region can comprise: the C-terminal subgroup one signature (COS), fibronectin type 3 (FN3), SPIa and the ryanodine receptor (SPRY), SPRY-associated domain (PRY), plant homeodomain (PHD), bromodomain (BROMO), filamin (FIL), NCL-1/HT2A/LIN-41 (NHL) repeats, meprin and tumor necrosis factor receptor-associated factor homology (MATH), acid-rich region (AR), ADP-ribosylation factor (ARF), and transmembrane (TM) domain. (**C**). TRIM proteins are RING-type E3 ligases that transfer ubiquitin (Ub) from the E2 enzyme to the substrate protein that is typically bound and recognized by the C-terminal variable region of the specific TRIM. (**D**). The structure of ubiquitin (adapted from PDB 1UBQ) with its seven internal lysine residues (K6, K11, K27, K29, K33, K48, and K63) that can potentially be conjugated to another Ub molecule via an isopeptide bond; this event then leads to oligo- or poly-ubiquitin chains with distinct linkages. Additionally, Ub can be linked through methionine 1 (M1) to another Ub molecule, which leads to the synthesis of M1-linked ubiquitination (also termed ‘linear’ ubiquitination). (**E**). Distinct poly-ubiquitin-linkage types as well as mono- or multi-mono-ubiquitination events exert specific effects on the modified substrate protein and thereby influence various cell biological processes, such as specific signal transduction pathways including immune signaling, proteasomal degradation, and autophagy (among others).

*Tripartite motif* (TRIM)-containing proteins, a family of post-translational modifiers, are present throughout the metazoan kingdom and expanded their family members (>10 members in flies; about 80 members in humans) during vertebrate evolution possibly due to the selection pressure posed by pathogen–host interactions. The prototypical TRIM protein carries a distinctive N-terminal tripartite RBCC motif containing a *R*eally *I*nteresting *N*ew *G*ene (RING) domain, one or two *B* Box domain(s), and a *c*oiled-*c*oil domain (*CC*D). The RBCC motif is then followed by C-terminal variable domain(s) such as PRY-SPRY, NHL, or ARF [21,22,23] (Figure 1). The highly organized domain architecture of TRIM proteins suggests that the functions of the individual domains are intricately synchronized. The zinc finger-containing RING domain possesses E3 ligase activity that covalently conjugates specific lysine residue(s) of a protein to ubiquitin (Ub), small ubiquitin-like modifier (SUMO), or the IFN-stimulated gene/protein of 15 kDa (ISG15) (as well as others) [24,25]. The CCD allows TRIM proteins to form higher-order homo- or hetero-oligomeric assemblies and coordinates the functional juxtaposition of other domains (e.g., RING and PRY-SPRY) facilitating TRIM-mediated substrate modification. Not surprisingly, certain viruses such as influenza A viruses (IAV) have evolved to target these specific CCD functions [26,27]. The variable domains at the C-terminus of TRIM proteins define their subcellular localization, enzymatic activity, protein substrate specificity, or nucleic acid-binding ability and are the basis for the classification of TRIM proteins into eleven specific subgroups (C-I to C-XI) [28,29] (Figure 1).

TRIM E3 ligases can catalyze mono-, oligo-, or poly-ubiquitination of a target protein, including another TRIM protein or ‘self’ (i.e., autoubiquitination). The specific poly-ubiquitin-linkage type attached to a particular lysine (K) residue has the potential to confer a distinct intracellular ‘signal’ or biological effect (Figure 1). Specific linkage types of ubiquitin chains catalyzed by TRIM proteins or other E3 ligases (and their reported functions) include K6 (DNA damage and proteasomal degradation), K11 (proteasomal degradation and cell cycle control), K27 (autophagy and signal transduction), K29 (signal transduction), K33 (signal transduction), K48 (proteasomal degradation), and K63 (immune signaling) [28,29]. As briefly mentioned above, some TRIM proteins also modify substrate proteins with ubiquitin-like (UBL) proteins such as ISG15 and SUMO, although these modifications are much less well studied for TRIM proteins.

Despite sharing the RBCC domain architecture, individual members of the TRIM protein family act on distinct substrates and thereby regulate a wide range of biological processes in health and disease contexts, including cell proliferation, autophagy, transcription, protein homeostasis, and cellular metabolism. Moreover, the role of TRIM proteins in modulating virus replication and infection outcome has been increasingly appreciated [28,29]. TRIM proteins can elicit antiviral activities primarily by (1) directly targeting viruses, (2) regulating virus infection-mediated cytokine responses, and/or (3) manipulating autophagy or apoptosis.

TRIM proteins can directly interfere with virus replication, and various mechanisms have been reported. Conceptually, TRIM proteins can prompt the proteasomal destruction of virions through antibody-mediated opsonization, or they can subject specific viral components to proteasome- or autophagy-mediated degradation. The direct antagonism of viral factors by TRIM E3 ligases has been studied in detail in the context of RNA virus infection [28,30]. Multiple TRIM proteins also intricately regulate the production of antiviral and proinflammatory cytokines or other virus-restricting molecules. Upon sensing of pathogen-associated molecular patterns (PAMPs) or danger-associated molecular patterns (DAMPs), host pathogen-recognition receptors (PRRs) initiate coordinated cascades of signaling events that lead to the expression of interferons (IFNs), including type I and type III IFNs, proinflammatory cytokines, and many chemokines. The type I IFN receptor (IFNAR) is eventually activated by secreted IFN-α/β, which, in an autocrine and paracrine manner, launches a cellular antiviral host response through the production of IFN-stimulated genes (ISGs). TRIM proteins have been demonstrated to finetune cytokine production by modulating either PRR-mediated signal transduction processes or signaling events downstream of cytokine receptors (e.g., IFNAR). For example, certain TRIM proteins directly modify PRRs with nondegradative ubiquitination (such as K63-linked ubiquitination), enhancing type I IFN gene expression. In contrast, the dampening of immune signaling is achieved by TRIM-mediated catalysis of degradative ubiquitination (e.g., K11- or K48-linked ubiquitination) of sensors or signaling mediators. Moreover, TRIM protein-mediated UBL modifications, such as SUMOylation of IFN-regulatory factor 7 (IRF7), also regulate the antiviral IFN response [21,28].

TRIM proteins also post-translationally modify proteins involved in autophagy or apoptosis, two biological host processes that play critical roles in regulating viral infection and pathogenesis [31]. Autophagy, classically recognized as a major cellular process for destruction of damaged organelles or unwanted proteins, can promote the replication of certain RNA viruses (e.g., flaviviruses), while it is well-known to restrict other viruses including HSV-1, human immunodeficiency virus 1 (HIV-1), and Sindbis virus [32,33]. Recent studies identified TRIM proteins as critical modulators of virus-induced autophagic responses, although the exact mechanisms by which specific TRIM proteins function in the autophagy process are still largely unknown [30]. Furthermore, host cells can utilize regulated cell death mechanisms (e.g., apoptosis) to eradicate infected cells, thereby restricting virus replication and spread. TRIM proteins have been shown to control apoptotic responses by regulating specific cell surface receptors or apoptosis ‘transducer’ molecules (as detailed below) [31].

### 1.3. Anti-Herpesvirus Immunity: Cytokine, Autophagy, and Apoptotic Host Responses

Innate immunological surveillance of human herpesvirus infection is achieved by various PRRs that recognize viral PAMPs or host-derived ligands (called DAMPs). The most well-studied herpesvirus-sensing pathways are (1) Toll-like receptors (TLRs) that patrol PAMPs at the plasma membrane and in endosomal compartments, as well as the cytosolic guardians; the (2) RIG-I-like receptors (RLRs) that sense RNA intracellularly; and (3) cyclic GMP-AMP synthase (cGAS), which senses cytosolic DNA [34,35,36,37]. Upon recognition of their ligands, PRRs activate downstream adaptor proteins such as Toll/interleukin-1 receptor domain-containing adaptor inducing IFN-β (TRIF), mitochondrial antiviral-signaling (MAVS), and stimulator of interferon genes (STING) to initiate the expression of chemokines, cytokines, and ISGs. The transcriptional response prompted by PRR activation is mediated by transcription factors such as IRFs, nuclear factor kappa-light-chain-enhancer of activated B cells (NF-κB), and others.

Cell surface-residing and endosomal PRRs such as TLR2 (recognizing glycoproteins), TLR3 (detecting dsRNA), TLR7/8 (recognizing ssRNA), and TLR9 (sensing unmethylated CpG motifs) detect herpesviruses in distinct cell types and then activate cytokine responses [34,38]. Cytosolic PRRs like RIG-I (recognizing blunt-ended dsRNA carrying a 5′-triphosphate moiety) and MDA5 (sensing long dsRNA) have been implicated in the initiation of type I IFN programs via MAVS and TANK-binding kinase 1 (TBK1). Molecular studies on RIG-I-mediated sensing of certain dsDNA viruses (i.e., HSV-1 and EBV) revealed that infection induces the translocation of the *5S* ribosomal RNA pseudogene 141 (*RNA5SP141*), a small noncoding RNA, from the nucleus to the cytoplasm, activating RIG-I and stimulating cytokine gene induction [39]. EBV-encoded small RNAs (EBERs) activate RIG-I, providing another line of evidence that RIG-I is a pivotal sensor for initiating DNA virus-induced innate immunity [40]. A role for MDA5 in the recognition of HSV-1 infection has also been reported; however, the molecular mechanism(s) of MDA5 activation by DNA viruses, including the specific ligands sensed by MDA5 in this context, remain elusive [35,41]. RIG-I binds to host-derived misprocessed noncoding RNAs (such as vault RNAs) during KSHV infection, and the reduced expression of dual-specificity phosphatase-11 (DUSP11, an RNA phosphatase) in KSHV-infected cells results in the accumulation of these triphosphorylated RNAs and thereby activation of RIG-I-mediated immune defense responses [42,43].

Among the various cytosolic or nuclear DNA sensors that detect HSV-1 and other herpesviruses, cGAS takes a central place. Upon activation, cGAS generates cGAMP (a secondary messenger), which initiates downstream signaling via the STING-TBK1-IRF3 axis to elicit type I IFN gene expression. To antagonize this pathway, HSV-1 and other herpesviruses have evolved multiple strategies. For instance, the HSV-1 protein ICP34.5 inhibits the translocation of STING from the endoplasmic reticulum (ER) to the Golgi, a critical signal transduction requirement. Moreover, the activated STING-TBK1 complex is hijacked by another HSV-1 protein, ICP27, dampening the subsequent signaling steps [35,44]. Similarly, KSHV-encoded ORF52 (an abundant gamma-herpesvirus-specific tegument protein) and its homologues in other herpesviruses evade cytosolic DNA sensing by directly inhibiting the enzymatic activity of cGAS [45]. Other cytosolic DNA sensors like AIM2 (absent in melanoma 2), ZBP1 (Z-conformation nucleic acid binding protein 1), and IFI16 (IFNγ inducible protein 16) also recognize herpesvirus infections and mount innate immune responses [37].

Herpesvirus infection also triggers specific cell death pathways (i.e., autophagy, apoptosis, and others). Autophagy is a vital cellular recycling mechanism that is designed to eliminate non-functional proteins, damaged organelles, and even pathogen components (e.g., viral proteins) by directing them to lysosomal degradation. In the context of herpesvirus infection, autophagy plays a critical role in limiting viral replication and virus-induced pathogenesis. For instance, central and peripheral murine neurons predominantly rely on autophagy to restrict HSV-1 infection [46]. In turn, HSV-1 has evolved strategies to evade the autophagy machinery by expressing specific antagonizing factors like γ34.5, US11, and ICP0 [47]. Similarly, other herpesviruses also encode proteins to subvert autophagic responses (e.g., v-BCL2 and v-FLIP of KSHV; BALF0 and BALF1 of EBV, and TRS1 of HCMV) [48,49,50].

Besides autophagy, the host organism employs apoptosis to control herpesviral replication. Apoptosis is a form of programmed cell death mediated by a group of cysteine protease enzymes, called caspases. Two types of activation modes exist for apoptosis. Intrinsic apoptosis is initiated following major changes or insults to the cellular microenvironment, such as DNA damage or mitochondrial outer membrane permeabilization. Extrinsic apoptosis is activated by distinct transmembrane receptors that are members of the tumor necrosis factor (TNF) receptor gene superfamily containing cysteine-rich extracellular domains and a cytoplasmic domain called the “death domain” [51]. HSV-1 uses multiple maneuvers to counteract apoptosis (i.e., by employing the viral proteins gD, gJ, US3, and LAT), which were summarized in other reviews [52]. Interestingly, HSV-1 has been shown to enhance apoptosis in neonatal neutrophils [53]. Like HSV-1, other herpesviruses such as HCMV, KSHV, and EBV utilize sophisticated mechanism(s) to inhibit apoptosis. For example, the HCMV vICA/pUL36 protein inhibits caspase-8-induced apoptosis [54].

## 2. TRIM Proteins Modulating PRR Responses to Herpesvirus Infection

**Figure 2:**
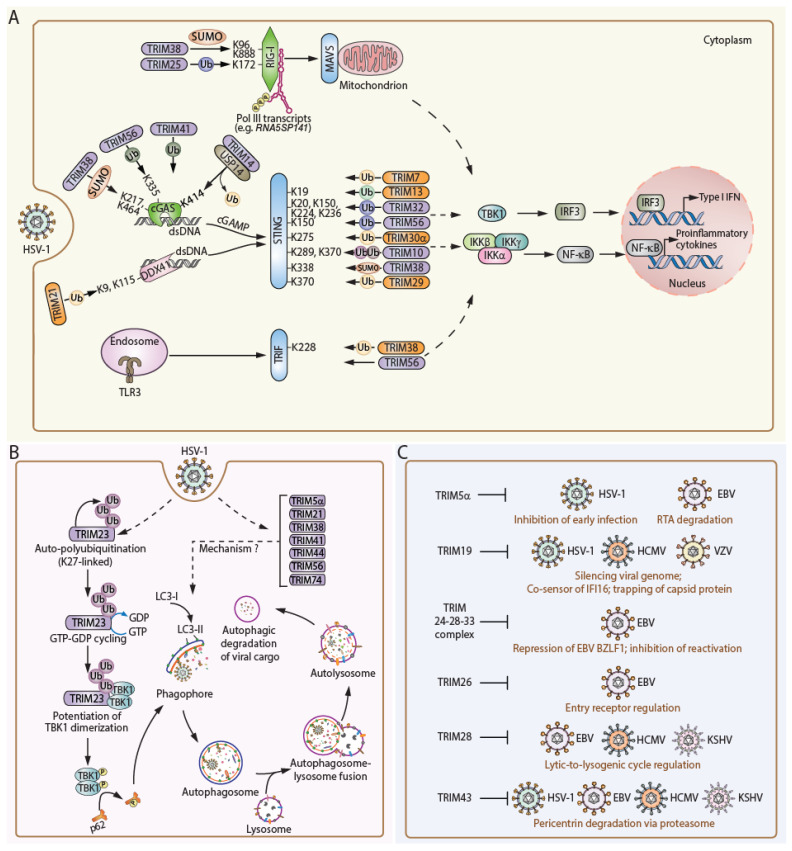
Mechanisms of TRIM protein-mediated regulation of herpesvirus infections. TRIM proteins utilize predominantly three distinct mechanisms to modulate herpesvirus infection: (**A**). Multiple TRIM proteins regulate the activity of specific pattern-recognition receptors (PRRs) or their adaptors involved in detecting HSV-1 infection and eliciting innate immune signaling via TBK1-IRF3 and IKK-NF-κB, thus modulating type I IFN and proinflammatory cytokine induction. Some TRIMs can also regulate the activity of molecules downstream of PRRs and adaptors (not illustrated). The different poly-ubiquitin-linkage types catalyzed by the respective TRIM proteins are illustrated in different colors following the scheme shown in Figure 1E. TRIM proteins illustrated in purple color are positive regulators of PRR signaling; TRIM proteins illustrated in orange color are negative regulators of PRR signaling. (**B**). Certain TRIM proteins orchestrate autophagy-dependent restriction of herpesvirus infection (e.g., HSV-1). HSV-1 infection triggers the E3 ligase activity of TRIM23, which leads to its K27-linked auto-polyubiquitination; this ubiquitination event is critical for the GTPase activity of the C-terminal ARF domain. The GTPase activity of the ARF domain potentiates the dimerization of TBK1, facilitating its activation by *trans*-autophosphorylation. Activated TBK1 phosphorylates p62, one of the key autophagy receptors that recognize viral cargos, promoting lysosome-dependent degradation. TRIM5α, TRIM21, TRIM38, TRIM41, TRIM44, TRIM56, and TRIM74 were identified to positively regulate HSV-1-induced LC3 (microtubule-associated protein 1 light chain 3) puncta formation; however, their precise mechanisms in autophagy regulation remain to be identified. (**C**). TRIM proteins can directly inhibit herpesviral replication through a variety of mechanisms. TRIM5α blocks EBV replication by inducing the degradation of a key viral component, the replication and transcription activator (RTA). TRIM19 restricts herpesvirus infection via multiple mechanisms: by silencing viral genomes as part of PML nuclear bodies, modulating IFN induction, and trapping viral capsid proteins. The TRIM24-TRIM28-TRIM33 complex represses EBV lytic infection by downregulating the expression of the EBV BZLF1 lytic ‘switch’ gene. TRIM26 binds to heat shock protein 90-beta (HSP-90β) and prompts its proteasomal degradation, influencing the integrity of EphA2 and thereby EBV entry. TRIM28 directly binds to the viral genome and regulates the lytic-to-lysogenic cycle. TRIM43 restricts multiple herpesviruses by ubiquitinating pericentrin, a centrosomal protein, triggering its proteasomal degradation, which ultimately induces changes in the nuclear lamina and thereby influences herpesvirus replication.

An array of TRIM proteins has been shown to regulate various PRR pathways that are important for sensing herpesvirus infection and mounting antiviral defenses. The innate immune response to HSV-1 (or other herpesvirus) infection is initiated primarily by the cGAS, RLR, and TLR signaling pathways (Figure 2). TRIM proteins were shown to regulate the activity of these sensor proteins, their adaptors (i.e., TRIF, MAVS, and STING), or common downstream molecules propagating immune signaling (e.g., TBK1 and IRF3). In most cases, TRIM E3 ligases catalyze specific PTMs on immune sensors or signaling proteins, reinforcing or dampening anti-herpesviral host defenses (Table 1).

### 2.1. cGAS-STING Signal Transduction

TRIM proteins regulate the cGAS-STING signaling pathway in various ways (Figure 2 and Table 1). A TRIM overexpression screen identified TRIM14 as a positive regulator of HSV-1-induced cGAS-STING signaling [58]. *TRIM14^−/−^* mice are highly susceptible to lethal HSV-1 infection due to impaired production of type I IFN. Mechanistically, cGAS earmarked with K48-linked poly-ubiquitin (at K414) undergoes p62-dependent autophagic degradation in HSV-1-infected cells, which is countered by TRIM14. TRIM14 recruits USP14, which then deubiquitinates cGAS leading to its sustained expression [58]. TRIM38 regulates the kinetics of the antiviral response to HSV-1 infection. In uninfected cells or early in HSV-1 infection, TRIM38 interacts with cGAS and STING and mediates their SUMOylation (cGAS at K217 and K464; STING at K338). These posttranslational modifications (PTMs) increase the protein stability of cGAS and STING potentiating immune signaling. Specifically, TRIM38-mediated SUMOylation of cGAS negatively regulates the K48-linked ubiquitination of cGAS and thereby protects it from proteasomal degradation. During the later phases of infection, the SUMOylation of cGAS and STING is removed by SENP2 (sentrin/SUMO-specific protease 2), leading to degradation of cGAS and STING by the proteasome and chaperone-mediated autophagy (CMA), respectively. At the molecular level, the non-SUMOylated pool of STING readily interacts with HSC70 (heat shock cognate 71 kDa protein), a receptor for CMA, whereas SUMOylation at K338 masks the HSC70-recognition motif (_326_QEVLR_330_) in STING, thus preventing its degradation [65].

Apart from regulating the protein stability of cGAS and STING, TRIM proteins also directly mediate the activation of cGAS-STING signaling. For example, TRIM41, also known as RINCK (RING-finger protein that interacts with C kinase), has been shown to catalyze the mono-ubiquitination and activation of cGAS during HSV-1 infection [66]. Knockdown of TRIM41 in HeLa cells not only impaired the activating phosphorylations of TBK1 and IRF3 but also dampened the production of cGAMP after dsDNA stimulation or HSV-1 infection [66]. A functional screen measuring IFN-β promoter-luciferase activation led to the identification of TRIM56 as a positive regulator of type I IFN responses to intracellular dsDNA. TRIM56 interacts with STING and catalyzes the K63-linked ubiquitination of STING at K150, thus promoting STING dimerization and the subsequent recruitment of TBK1 [68]. In an independent study, TRIM56 has been shown to also act on cGAS [67]. TRIM56 binds to cGAS and catalyzes the mono-ubiquitination of the sensor at K335, facilitating its dimerization and enhancing its dsDNA-binding activity. WT but not *TRIM56*^−/−^ bone marrow-derived macrophages (BMDMs) infected with either WT HSV-1 or HSV-1ΔICP34.5 readily produced IFNα/β and restricted virus infection [67]. However, when infected with RNA viruses (e.g., dengue, IAV, or Zika virus), both WT and *TRIM56*^−/−^ BMDMs produced similar levels of IFNβ transcript, suggesting that TRIM56 primarily regulates innate immunity to DNA viruses, but not RNA viruses [67]. The ubiquitin regulatory X domain-containing protein 3B (UBXN3B) has been shown to interact with STING and its upstream E3 ligase TRIM56 to facilitate STING ubiquitination, dimerization, trafficking, and downstream signaling via TBK1 [69]. Like *STING*^−/−^ mice, *UBXN3B*^−/−^ adult mice are highly susceptible to HSV-1 infection. HSV-1 infection induced the formation of a TRIM56-STING-UBXN3B ternary complex, facilitating the K63-linked ubiquitination of STING. Another TRIM protein reported to boost STING signaling is TRIM10. Upon HSV-1 infection, TRIM10 interacts with the c-di-GMP binding domain of STING; this event then induces the K27- and K29-linked ubiquitination of STING (at K289 and K370), leading to enhanced STING aggregation at the Golgi apparatus and, thereby, its activation. Confocal microscopy imaging analysis assessing the colocalization of phosphorylated (activated) TBK1 with STING showed that WT STING, but not its K370R mutant, colocalized with phospho-TBK1 at the Golgi following ISD (IFN stimulating DNA) treatment [56]. Another TRIM protein, TRIM32, acts at the step of STING-TBK1 complex formation. Upon virus infection, TRIM32 translocates from the cytosol to mitochondria and the ER where it complexes with STING to carry out K63-linked polyubiquitination of STING (at K20/150/224/236), which facilitates the STING-TBK1 interaction [64].

A few TRIM proteins also dampen cGAS-STING signaling. TRIM7 (also known as RING-finger protein 90, RNF90) earmarks STING with K48-linked poly-ubiquitin, leading to the proteasomal degradation of STING, which eventually quenches innate immune signaling [55]. *TRIM7*^−/−^ mice infected with HSV-1 showed a better survival compared to infected WT mice. Similarly, *TRIM13*^−/−^ BMDMs and mouse embryonic fibroblasts were reported to elicit enhanced cytokine production upon HSV-1 infection compared to WT cells [57]. Mechanistically, TRIM13 (via its transmembrane domain) interacts with STING at the ER and facilitates the K6-linked ubiquitination of STING (at K19), blunting STING dimerization and ER retention. As a result, STING undergoes ER-initiated degradation [57]. Similarly, TRIM29 promotes DNA virus infection (e.g., HSV-1 and EBV) by inhibiting cGAS-STING signaling. Mice lacking TRIM29 are resistant to HSV-1 due to enhanced production of type I IFN [62]. At the molecular level, transcriptional induction of TRIM29 (which is an ISG) facilitates the K48-linked ubiquitination of STING at K370; this PTM mark promotes STING degradation, thereby negatively regulating antiviral defenses against HSV-1 and EBV [62]. Another TRIM protein whose deficiency augments cytokine responses to pathogenic DNA exposure is TRIM30α. TRIM30α catalyzes STING ubiquitination at K275, triggering its destruction by the proteasomal machinery [63]. As a consequence, *TRIM30α^−/−^* mice are more resistant to HSV-1 infection.

Besides cGAS, various other intracellular DNA sensors that regulate immunity to herpesviruses have been identified [79,80,81]. One of the major dsDNA sensors expressed in myeloid dendritic (and other) cells is Dead-box helicase 41 (DDX41), which mediates type I IFN responses via STING. Co-immunoprecipitation of DDX41 followed by mass spectrometry analysis identified TRIM21 as an interacting partner [60]. Knockdown of TRIM21 in THP-1 (human monocytic) cells followed by HSV-1 infection enhanced the production of IFNβ, suggesting a role for TRIM21 in dampening type I IFN responses. Molecular studies identified TRIM21 as a direct E3 ligase for DDX41 mediating its K48-linked ubiquitination at K9 and K115. As a result, DDX41 is degraded [60].

### 2.2. RLR Signaling

Although HSV-1 is a dsDNA virus, RLR RNA helicases have emerged as important sensors of HSV-1 infection [39,82]. Several RNA ligands that either originate from the virus or are produced by the host cell (i.e., certain RNA polymerase III (Pol III) transcripts) can bind and activate RIG-I during herpesvirus infection [39,83]. Next-generation sequencing of RNAs bound to RIG-I during HSV-1 infection identified the *5S* ribosomal pseudogene transcript *RNA5SP141* (a 120 nucleotide-long noncoding RNA) as a RIG-I host-cellular ligand [39]. Mutations in the *GTF3A* gene, which encodes for transcription factor IIIA (TFIIIA), a Pol III co-factor needed for *RNA5SP141* transcription, can predispose humans to HSE [82]. *RNA5SP141* is transcriptionally upregulated in response to HSV-1 infection and mislocalizes to the cytoplasm where it engages with RIG-I. Specific *RNA5SP141*-interacting proteins (i.e., mitochondrial ribosomal protein L18 (MRPL18) and thiosulfate sulfurtransferase (TST)) prevent RIG-I activation by shielding or ‘masking’ *RNA5SP141* in uninfected cells. However, infection with HSV-1 and other herpesviruses (e.g., EBV) enhances the accessibility of this cellular RNA ligand to RIG-I by inducing the degradation of MRPL18 and TST. Besides ‘masking’ of host cellular immunostimulatory RNAs, RLR activation is prevented in uninfected cells by Ser/Thr phosphorylation of their CARD domains, which relay the signaling of RLRs to MAVS [84]. Dephosphorylation of RLRs mediated by the PPP1R12C-PP1α/γ phosphatase complex and their subsequent “PTM earmarking” by ubiquitin or UBL modifications (i.e., K63-linked ubiquitination of RIG-I by TRIM25 and Riplet; ISGylation and K63-linked ubiquitination of MDA5 by HERC5 and TRIM65, respectively) facilitate RLR oligomerization and signaling [61,84,85,86,87]. In addition to K63-linked polyubiquitination, RIG-I has been shown to require SUMOylation (at K96 and K888) for its optimal activation, which is catalyzed by TRIM38 [25] (Figure 2). As expected, many viruses have developed ways to antagonize some of these key PTM events driving RLR activation [88]. In regard to herpesviruses, the HSV-1 kinase US3 interacts with RIG-I and phosphorylates it at S8; this then inhibits several RIG-I activation steps such as TRIM25-mediated ubiquitination and the complex formation with MAVS [89]. A recombinant HSV-1 encoding a catalytically dead (K220A) US3 kinase mutant loses its ability to phosphorylate RIG-I and hence induces a heightened expression of ISGs and cytokines. Interestingly, this RIG-I-inhibitory mechanism is conserved for alphaherpesvirus US3 kinases [89]. The large tegument proteins of herpesviruses encode a conserved cysteine protease with ubiquitin-specific deconjugase activity. The HSV-1 deconjugase UL36 and the EBV homologue BPLF1 promote TRIM25 autoubiquitination, which ultimately antagonizes TRIM25-mediated RIG-I ubiquitination and activation. Such a mechanism of disruption of TRIM25-mediated RIG-I activation by viral deconjugases is conserved among several herpesviruses [90].

### 2.3. TLR Signaling

TLRs recognize a variety of PAMPs and are pivotal for the immune surveillance of herpesvirus infection. Especially TLR3, which mediates downstream signaling via TRIF, has been extensively investigated in the context of herpesvirus infection [34]. Recent studies identified a few TRIM proteins that regulate the immune responses elicited by TLR3 [91]; notably, their roles have been characterized mostly in other contexts (not herpesvirus infection). For example, the C-terminus of TRIM56 interacts with TRIF potentiating TLR3 signaling in an E3 ligase activity-independent manner [92]. TRIM38 negatively regulates the cytokine induction elicited by TLR3 (and also TLR4) via two distinct mechanisms [93]. First, TRIM38 interacts with TRIF and catalyzes its K48-linked polyubiquitination (at K228), accelerating its proteasomal degradation in immune cells. Second, TRIM38 is an ISG and, upon its transcriptional upregulation, negatively regulates TNFα and IL-1β signaling specifically in IFN-primed immune cells by degrading TAB2 (TGFβ-activated kinase 1 binding protein 2) via autophagy. While the specific role of TRIM38 in the context of HSV-1 infection has not yet been elucidated, it may be possible that TRIM38 activity is harmful during the early stage of infection due to its dampening effect on cytokine responses, whereas it may play a beneficial role during the late phase of infection or in a chronic infection setting by preventing excessive inflammatory responses.

## 3. TRIM Proteins Regulating Autophagy and Apoptosis

### 3.1. Autophagy

Recent studies indicated that TRIM proteins regulate various steps of autophagy, including the initiation of autophagy, cargo capture and delivery, and autophagosome formation [94,95]. Some TRIM proteins enable the autophagic degradation of innate immune sensors or signaling molecules, which also impacts antiviral responses. The molecular details of how TRIM proteins regulate autophagy in the context of herpesviral infection remain largely elusive [70,94]. A combined TRIM cDNA and siRNA screening approach identified TRIM23 as a critical regulator of autophagy induced by HSV-1, IAV, and encephalomyocarditis virus (EMCV) [70]. In the context of HSV-1 infection, silencing of TRIM5α, TRIM21, TRIM38, TRIM41, TRIM44, TRIM56, and TRIM74 diminished autophagy; however, the exact mechanisms through which these TRIMs apparently promote autophagic responses to HSV-1 are currently unknown (Figure 2 and Table 1). In contrast, the molecular details by which TRIM23 promotes autophagy and HSV-1 restriction have been recently defined. TRIM23 catalyzes the K27-linked autoubiquitination of its ARF domain, which triggers TRIM23′s GTPase activity. HSV-1-induced TRIM23 activation facilitates the multimerization and *trans*-autophosphorylation of TBK1, the latter mediates the phosphorylation of the autophagy receptor p62 (at S403). Autophagy elicited by TRIM23 effectively restricts HSV-1 replication, likely through lysosomal degradation of HSV-1 components [70] (Figure 2). Interestingly, TRIM23 has also been shown to interact with UL144 of HCMV and to mediate NF-κB activation during HCMV infection [96]. TRIM31 promotes ATG5- and ATG7-independent autophagy in intestinal cells and is downregulated by HCMV infection [97]. However, the involvement of these TRIM members in autophagy regulation during other herpesvirus infections is currently unknown. Notably, many TRIMs also regulate non-viral autophagy [98] or act as autophagy cargo receptors in certain contexts [99]. It remains to be elucidated whether any of these TRIM proteins exert similar functions in autophagy during herpesvirus infection.

### 3.2. Apoptosis

Besides modulating autophagy, several TRIM proteins are known to regulate apoptosis and cell death, which have been well studied in cancer [31]. The first TRIM protein identified to cause apoptosis when overexpressed was TRIM19, which induces caspase-independent and p53-dependent apoptosis [100]. The pyrin domain of TRIM20 interacts with ASC (apoptosis-associated speck-like protein containing a CARD domain), which eventually activates caspase 1 and causes apoptotic cell death [101]. Similarly, TRIM32 interacts with X-linked inhibitor of apoptosis (XIAP), a negative regulator of apoptosis, and targets it for degradation, hence sensitizing cells to TNFα-induced apoptosis [102]. TRIM32 expression is upregulated by KSHV during latency, whereas TRIM32 undergoes degradation via the proteasomal machinery during the lytic infection phase. Such inhibition of TRIM32 expression induces cellular apoptosis which, in turn, inhibits the proliferation of KSHV-infected primary effusion lymphoma (PEL) cells [103]. TRIM35 acts as a tumor suppressor in breast cancer and mediates ubiquitination and hence degradation of PDK1 (3-phosphoinositide-dependent protein kinase-1), ultimately leading to apoptosis [104]. In contrast, TRIM27 acts as an anti-apoptotic protein by directly interacting with IκBα, a protein negatively regulating NF-κB, and promotes cell proliferation in renal cancer [105]. Of note, the specific roles of TRIM27, TRIM35, and various other TRIM proteins in regulating apoptosis during herpesvirus infection remain to be determined.

## 4. TRIM Proteins Directly Regulating the Herpesviral Life Cycle

To date, only a few TRIM proteins have been shown to directly target herpesviral factors or interfere with key steps of the herpesviral replication cycle (Figure 2 and Table 1). The simian TRIM5α protein can restrict HSV replication at the early stages of HSV infection, and it is speculated that HSV can overcome this restriction by reducing the expression levels of TRIM5α [106]. During EBV infection, TRIM5α reportedly acts as an E3 ligase for the replication and transcription activator (RTA) protein, promoting its proteasomal degradation; this event then attenuates the lytic cycle progression [71]. In addition, TRIM5α can induce the ubiquitination of BORF1 (an EBV capsid protein), destabilizing BORF1 by promoting its autophagic degradation [107]. TRIM24 and TRIM33 have been shown to target BZLF1 (a EBV lytic switch gene), thus suppressing EBV reactivation [75]. The phosphorylation of TRIM28 mediated by ataxia telangiectasia mutated (ATM) or mammalian target of rapamycin (mTOR) promotes the reactivation of multiple herpesviruses including EBV and HCMV [108,109]. The recruitment of TRIM28, together with heterochromatin protein 1 (HP1) and the histone methyltransferase SETDB1, to the viral genome has been shown to result in transcriptional gene silencing that promotes HCMV latency. In contrast, the activity of TRIM28 is suppressed by phosphorylation during lytic HCMV infection [109]. Furthermore, the KSHV-encoded protein LANA (latency-associated nuclear antigen) recruits TRIM28 to the viral genome to repress lytic gene expression during early infection [77].

TRIM19, also known as promyelocytic leukemia (PML) protein, is a key component of the dynamic subnuclear structures called PML nuclear bodies (PML-NBs). These macromolecular structures were first identified as nuclear dots and named nuclear domains 10 (ND10) based on their average number of 10 loci per nucleus in cultured cells. Initial observations indicating that the size and numbers of ND10 are decreased in HSV-1-infected cells led to defining the role of ND10 in herpesvirus infection. To date, the significance of PML and other ND10 components in modulating the replication of herpesviruses (and other viruses) has been well documented (reviewed extensively elsewhere [73,110,111]). PML together with other ND10 components elicits intrinsic anti-herpesviral defense via at least three mechanisms: (1) epigenetic regulation of viral gene expression, (2) physical entrapment of viral proteins, and (3) regulation of innate cytokine responses. Immediately after their nuclear entry, the genomes of herpesviruses (e.g., HSV-1 and HCMV) interact with PML-NBs, which results in epigenetic silencing of viral gene expression. Interestingly, in the context of VZV infection, PML-NB ‘cages’ are formed that physically entrap newly assembled viral nucleocapsids, resulting in the impairment of their nuclear egress [74]. Recently, an important role for ND10 components in the regulation of innate cytokine responses has emerged. These investigations were prompted by the observation that the expression of PML/TRIM19 (and several other ND10 components) is IFN inducible, and, further, that IFN-mediated antiviral responses are blunted in PML/TRIM19-deficient cells. Several studies have established a key role for PML/TRIM19 in IFN induction and downstream ISG responses, and multiple mechanisms have been proposed [73]. For example, PML/TRIM19 has been shown to regulate the sensing of HSV-1 dsDNA by the immune receptor IFI16 and the subsequent activation of antiviral immunity [59]. Besides these well-recognized functions in anti-herpesviral defense, PML/TRIM19 and ND10 components have also been shown to exert proviral effects [111].

Some TRIM proteins inhibit herpesvirus replication by altering the nuclear lamina or by repressing viral entry. TRIM43, which was identified in a TRIM shRNA screen for modulating KSHV reactivation in cultured cells, is a restriction factor for multiple herpesviruses including HSV-1, KSHV, HCMV, and EBV. TRIM43 blocks herpesvirus lytic replication by ‘earmarking’ pericentrin, a key protein of centrosomes, with K48-linked polyubiquitin; this ubiquitination event triggers pericentrin’s proteasomal degradation, causing altered nuclear lamina structures and thereby repressing lytic replication [78]. TRIM43 is unique in that it is expressed at very low levels in uninfected cells; however, after herpesvirus infection, TRIM43 transcript and protein levels are drastically upregulated, which is mediated by the transcription factor DUX4 (Double Homeobox 4) [78]. TRIM26 suppresses EBV infection by targeting Eph receptor A2 (EphA2), a key receptor for EBV entry into epithelial cells [76]. Mechanistically, TRIM26 induces poly-ubiquitination of heat shock protein 90-beta (HSP-90β), triggering its proteasomal degradation. This degradation event influences the integrity of EphA2 and thereby restricts EBV entry into cells [76].

## 5. Herpesviral Proteins Antagonizing TRIM Proteins

Despite extensive investigation into the sophisticated mechanisms by which TRIM proteins modulate herpesvirus replication, only little is still known about how herpesviruses antagonize individual TRIM proteins. One of the prominent examples of TRIM antagonism is the inhibition of TRIM19 and ND10 components by multiple herpesviruses. The ICP0 protein of HSV-1 acts as a SUMO-targeted ubiquitin ligase (STUbL) inducing the proteasomal degradation of several SUMO-conjugated proteins, including TRIM19 [112]. The immediate early protein IE1 of HCMV binds to TRIM19 and disrupts its SUMOylation leading to dispersal and inactivation of ND10 structures. Mechanistically, the globular core domain of IE1 (IE1core), which shares secondary structure features with the conserved CCD of TRIM proteins, binds to TRIM19 via coiled-coil interactions; this event then inhibits TRIM19 SUMOylation [113,114]. Gammaherpesviruses also antagonize PML/TRIM19 and ND10 utilizing their conserved tegument proteins. For example, the ORF75c protein of murine gammaherpesvirus 68 (MHV-68) induces the proteasomal degradation of PML [115].

TRIM23, a central component of virus-induced autophagy, is antagonized by HSV-1 US11 [116]. Specifically, US11 binds to the C-terminal ARF domain of TRIM23 that is critical for TRIM23′s GTPase activity as well as for mediating TRIM23′s interaction with TBK1. US11 binding derails the TBK1-TRIM23 complex, thereby overcoming autophagy-induced virus restriction [116]. Moreover, HSV-1 virulence factor γ_1_34.5 interacts with and stabilizes ICP0 (an immediate early protein synthesized post HSV-1 infection) by protecting it from TRIM23-induced degradation (mediated by K11- and K48-linked ubiquitination events) [117]. Mechanistically, the γ_1_34.5 protein prevents TRIM23′s autoubiquitination process and thereby its ICP0-targeting activity [117]. Interestingly, ICP0 is an E3 ligase itself and has a multitude of functions and substrates. Among its various targets is TRIM27, which is degraded by ICP0 via the proteasome, ultimately promoting HSV-1 infection [118]. The BZLF1 protein of EBV has been shown to interact with TRIM24 and TRIM33; these interactions prompt the degradation of these TRIM proteins, resulting in the disruption of TRIM24-TRIM28-TRIM33 complexes that function to suppress EBV reactivation [75]. In addition, RTA, the master regulator of KSHV lytic replication, interacts with TRIM32 and promotes its degradation via the proteasome system, ultimately facilitating KSHV reactivation [103]. Future studies are warranted to investigate the molecular mechanisms by which other herpesviral proteins counteract specific TRIM proteins or, alternatively, hijack the activity of TRIM proteins in infected hosts to promote optimal viral replication and/or to establish latency.

## 6. Future Perspectives

TRIM proteins have been implicated in various antiviral processes, including direct pathogen restriction and the regulation of innate immunity. Work from many groups has integrated our understanding of the intrinsic properties that govern the enzymatic or non-enzymatic activities of individual TRIM proteins. However, much less is currently known about the regulation of the intrinsic activities of TRIMs within cells. Important questions that remain unanswered include how the enzymatic activities and protein–protein interactomes of individual TRIM proteins are regulated during specific viral infections or in different cell types. Further, it is still largely elusive how the activity of TRIM proteins is dampened in uninfected conditions or after the resolution of the infection. While the role of TRIM proteins in regulating innate immune activation is well appreciated, whether TRIM proteins orchestrate adaptive immune defenses against viral infection is not known. Recent studies identified novel activities for some TRIMs, including their binding to RNA species [119,120,121]. As such, understanding the role of RNA-TRIM protein interactions in pathogen infection and immune homeostasis is a significant area of future research. Whether certain TRIMs can bind viral RNA produced by HSV-1 (or other DNA viruses), or perhaps even viral DNA, is another interesting avenue of investigation.

While most of our knowledge about TRIM functions is from emerging RNA virus infections, there is still a limited understanding of the relevance of TRIM proteins in restricting or supporting DNA virus infection, especially non-herpesviruses (like hepatitis B virus, poxviruses, and polyomaviruses). Along these lines, many strategies to antagonize or utilize specific TRIM proteins have been uncovered for RNA viruses; however, our knowledge of how DNA viruses dysregulate or usurp TRIM enzymes is largely unknown [122]. Moreover, the in vivo relevance of many TRIM proteins in modulating herpesviral pathogenesis is still unknown, and the generation and testing of additional TRIM-deficient mouse models are expected to greatly advance the field.

TRIM proteins have been associated with several types of human malignancies, but it remains to be uncovered how specific TRIM activities influence the oncogenesis induced by EBV and KSHV of the gamma-herpesvirus subfamily or other tumor viruses, including HBV, hepatitis C virus, and human papillomaviruses. Along these lines, given the central role of TRIM proteins in orchestrating proinflammatory host response programs, future studies should focus on defining the role of TRIM proteins in aging processes, including neurodegenerative diseases (e.g., AD), which are driven by excessive inflammation and have been linked to certain herpesvirus infections.

## Figures and Tables

**Table 1:** TRIM proteins regulating herpesvirus infection and their mechanisms of action.

TRIM	Target(s) and Molecular Mechanism(s)	Biological Effect	Reference
A. TRIM proteins that modulate PRR responses			
TRIM7	K48-linked ubiquitination of STING; proteasomal degradation	Dampening innate immune signaling	[55]
TRIM10	K27- and K29-linked ubiquitination of STING (at K289 and K370, respectively); activation	Promoting innate immune signaling	[56]
TRIM13	K6-linked ubiquitination of STING (at K19); proteasomal degradation	Dampening innate immune signaling	[57]
TRIM14	Recruitment of USP18 to promote removal of K48-linked poly-ubiquitin from cGAS (at K414); inhibition of degradation	Promoting innate immune signaling	[58]
TRIM19	Co-sensor of IFI16; detection of viral DNA	Promoting innate immune signaling	[59]
TRIM21	K48-linked ubiquitination of DDX41 (at K9 and K115); proteasomal degradation	Dampening innate immune signaling	[60]
TRIM25	K63-linked ubiquitination of RIG-I (at K172); activation	Promoting innate immune signaling	[39,61]
TRIM29	K48-linked ubiquitination of STING (at K370); proteasomal degradation	Dampening innate immune signaling	[62]
TRIM30α	K48-linked ubiquitination of STING (at K275); proteasomal degradation	Dampening innate immune signaling	[63]
TRIM32	K63-linked ubiquitination of STING (at K20, K150, K224, and K236); activation	Promoting innate immune signaling	[64]
TRIM38	SUMOylation of cGAS (at K217 and K464) and STING (at K338); activation	Promoting innate immune signaling	[65]
TRIM41	Mono-ubiquitination of cGAS; activation	Promoting innate immune signaling	[66]
TRIM56	Mono-ubiquitination of cGAS (at K335); activation	Promoting innate immune signaling	[67]
TRIM56	K63-linked ubiquitination of STING (at K150); activation	Promoting innate immune signaling	[68,69]
B. TRIM proteins that regulate herpesvirus-induced autophagy			
TRIM23	K27-linked auto-ubiquitination of its ARF domain; activation of TRIM23′s GTPase; facilitating TBK1- and p62-mediated autophagy	Promoting autophagy and HSV-1 restriction	[70]
TRIM5α, 21, 38, 41, 44, 56, 74	Unknown	Promoting HSV-1-induced autophagy	[70]
C. TRIM proteins that directly regulate the herpesvirus life cycle			
TRIM5α	Ubiquitination of EBV RTA protein; proteasomal degradation	Inhibition of EBV lytic replication	[71]
TRIM19	Epigenetic silencing of herpesviral genomes; repression of herpesviral protein expression; entrapment of capsid proteins	Inhibition of herpesvirus replication (HSV-1, HCMV, EBV, KSHV, VZV)	[59,72,73,74]
TRIM24-28-33 complex	Repression of EBV BZLF1; inhibition of lytic gene expression	Suppression of EBV reactivation	[75]
TRIM26	Ubiquitination and degradation of HSP-90β; regulation of EphA2 integrity	Inhibition of EBV entry	[76]
TRIM28	Interaction with KSHV LANA protein; inhibition of lytic gene expression	Promoting KSHV latency establishment	[77]
TRIM43	K48-linked ubiquitination and degradation of pericentrin; alteration of nuclear lamina architecture	Inhibition of herpesvirus replication (HSV-1, HCMV, EBV, KSHV)	[78]
